# Pupillometry in the Emergency Department: A Tool for Predicting Patient Disposition

**DOI:** 10.5811/westjem.39912

**Published:** 2025-07-07

**Authors:** Hector Gonzalez, Yanying Chen, Newton Addo, Debbie Y. Madhok

**Affiliations:** * Stanford School of Medicine, Palo Alto, California; † University of California San Francisco School of Medicine, San Francisco, California; ‡ University of California San Francisco, Department of Emergency Medicine, San Francisco, California; § University of California San Francisco, Department of Neurology, San Francisco, California

## Abstract

**Introduction:**

The ability to accurately assess and predict the disposition of comatose patients from within the emergency department (ED) remains a critical challenge. Traditional methods lack precision and consistency. Our goal was to evaluate the prognostic capability of the neurological pupil index (NPI) in predicting patient disposition from within the ED.

**Method:**

This prospective observational study followed 50 comatose patients (Glasgow Coma Scale [GSC] score < 9) who were enrolled via convenience sampling and subsequently treated in the ED at a Level 1 trauma center and public safety-net hospital in San Francisco, CA. We calculated NPI scores and collected data on patient demographics, clinical characteristics, and outcomes. The NPI scores were categorized into three groups: 0 (very poor); 0.1–3.0 (poor to moderate); and 3.1–5.0 (good). We used ANOVA, the Pearson chi-squared test, Wilcoxon rank-sum test, and Fisher exact test to assess the association between NPI scores and discharge status. Results were reported as odds ratios with 95% confidence intervals, with a *P*-value < .05 considered statistically significant.

**Results:**

The median age of patients in this study was 58 years (IQR 42–74), and 66% were male. Higher NPI scores (five-point scale with 3.1–5.0 considered normal) were significantly associated with an increased likelihood of ED discharge (82%), , while lower NPI scores (0, nonreactive pupil) were predominantly associated with hospital admission (92%) (P < .001). Significant predictors of discharge status included patient age, GCS scores, and coma etiology.

**Conclusion:**

This study highlights the utility of the NPI in predicting patient disposition from within the ED. Higher NPI scores were strongly associated with an increased likelihood of ED discharge. These findings support the idea that NPI has the potential to enhance the accuracy of prognostic assessments, in comparison to subjective characterizations of pupil activity. Additional research with larger, multicenter cohorts are needed to confirm these results and establish standardized protocols for integration of NPI in ED workflow.

## INTRODUCTION

The ability to accurately assess and prognosticate the disposition of comatose patients from within the emergency department (ED) remains a critical challenge. Traditional methods, such as manual pupillary light reflex (PLR) assessments, lack precision and consistency.^1^ Recent advancements in automated, quantitative pupillometry provide a more reliable approach to measuring neurological function made possible by the advent of the neurological pupil index (NPI).^2^ While it is important to note that this tool should not be used in isolation, its utility as a clinical adjunct offers a promising means of evaluating patients with low Glasgow Coma Scale (GCS) scores, especially in cases involving drug overdoses, where rapid and accurate assessment is crucial for determining outcomes and guiding clinical decisions.^3^,^4^

For patients who are comatose, it is crucial that emergency clinicians have the ability to accurately prognosticate health outcomes. These assessments can guide critical decision-making processes, such as determining whether a patient should be discharged or admitted for further workup. Accurate prognostication ensures that patients receive the appropriate level of care, ultimately impacting their recovery, and can guide clinical decision-making to avoid poor health outcomes. For comatose patients, traditional prognostic methods of pupil assessment often rely on subjective characterizations such as “brisk” and “sluggish,” which may lead to variability in clinical decisions.^5^ The NPI offers an objective, reliable measure that can enhance the accuracy of these prognostic assessments and provides a standardized metric that is less susceptible to interobserver variability.^6^,^7^

The rising incidence of drug overdoses, particularly those involving opioids and combinations with other substances like methamphetamine and cocaine, has intensified the need for reliable diagnostic tools in the ED. The city of San Francisco has seen a significant increase in overdose deaths, primarily driven by fentanyl and its combination with other drugs.^8^ The complex and negative effects on health caused by these drugs necessitate advanced methods to accurately differentiate between drug effects and underlying neurological conditions. Studies have demonstrated the robustness of the NPI even in the presence of significant drug-induced miosis and other ocular effects, supporting its use as a stable measure of neurological function across an array of intoxication scenarios.^9^,^10^

Most of the currently available literature on the NPI is based in intensive care unit (ICU) settings, where it has demonstrated utility in mapping the trajectory of neurological function. Research has shown that the NPI has been effective in the assessment of traumatic brain injury, predicting outcomes in patients with intracerebral hemorrhage and aiding in the prognostication of comatose patients following out-of-hospital cardiac arrest.^5^,^11^ While NPI applications have been well-documented in ICU populations, our aim in this study was to investigate the effectiveness and utility of this tool in predicting patient discharge or admission for further workup in comatose patients presenting to the ED. We hypothesized that higher NPI scores would correlate with increased likelihood of discharge. By correlating NPI measurements with clinical outcomes, we sought to validate the NPI as a reliable tool for early neurological assessment and decision-making from within the ED.

## METHODS

### Study Design

This prospective observational study was designed to evaluate the effectiveness of the NPI in predicting patient disposition. We obtained institutional review board approval from our institution (IRB number 22-38188) and received funding in research grant support from NeurOptics. As each comatose patient automatically had a pupillary assessment at the time of the study, the need to obtain consent was waived per the IRB.

**Population Health Research Capsule**

What do we already know about this issue?

*Quantitative pupillometry has shown prognostic value in ICU settings but is understudied in emergency department populations*.

What was the research question?

*Can the neurological pupil index (NPI) predict ED disposition in comatose patients?*

What was the major finding of the study?

*Higher NPI scores were significantly associated with ED discharge (P < .001; 95% CI 1.3–4.2)*.

How does this improve population health?

*Objective pupillometry may aid emergency physicians in disposition decisions, optimizing resource use and reducing unnecessary admissions*.

For the purposes of this study, we defined coma as a GCS ≤8, consistent with the Neurocritical Care Society definition. This threshold represents a severe impairment of consciousness, where patients exhibit limited or absent meaningful responses to external stimuli. While different clinical interpretations of coma exist, this definition allows for a standardized approach to identifying patients with significant neurological dysfunction requiring urgent assessment and management.

### Study Setting and Population

From June 2023–February 2024, we conducted this study at the Zuckerberg San Francisco General Hospital, a public, safety-net hospital and Level I trauma center in the city of San Francisco, CA. Participants were enrolled via convenience sampling, given that pupillometer measurements were not mandated by protocol and were instead obtained at the discretion of the individual performing the assessment. Since this was not a universally applied assessment, the total number of enrolled patients was determined by the frequency with which clinicians opted to use the device rather than a predefined sample-size calculation.

The inclusion criteria for the study were adult patients ≥18 years of age who presented to the ED with a GCS score of <9 and had a comatose state of presumed non-traumatic etiology. Patients in cardiac arrest or pulseless on arrival were not automatically excluded from the study. However, we included in the analysis only those who achieved return of spontaneous circulation at some point during their ED course, as we aimed to assess neurological outcomes and disposition rather than immediate resuscitation outcomes. Patients who received a pupillometer measurement upon ED arrival were included. Exclusion criteria were patients <18 years of age and those with coma resulting from traumatic brain injury or eye injury precluding the use of pupillometry.

### Protocol

The NPi-200 pupillometer (NeurOptics Inc, Irvine, CA) is a handheld, portable infrared device designed to provide objective, quantitative measurements of pupillary response. Roughly the size of a barcode scanner, the device is placed close to the eye where it emits a controlled light stimulus while recording high-resolution video of the pupil’s reaction that can be seen in real time by the user through a screen. The device automatically calculates multiple pupillary parameters including pupil size prior to constriction, minimum diameter at peak constriction, latency of constriction, constriction velocity, and dilation velocity.

The personnel tasked with obtaining pupillometer measurements included emergency physicians, nursing staff, a clinical research coordinator, and a medical student. All device users underwent standardized training on the proper handling and operation of the pupillometer consisting of an instructional video.

Upon presentation to the ED, each patient meeting the inclusion criteria underwent a pupillometer measurement as part of the standard ED resuscitation protocol. Pupillometer measurements were conducted using the NPi-200 pupillometer with a single-use safety guard to ensure sterility and tagged with a specific identification number. We then linked these measurements to the patient’s medical record number via electronic health records (Epic Systems Corporation, Verona, WI); physiology data (Moberg CNS System (Natus Medical Inc, Pleasanton, CA); electroencephalography (Natus); and radiology (Picture Archiving and Communication System).

This study was funded by NeurOptics; however, the manufacturer had no role in the study design, data collection, data analysis, or decision to publish the findings. Additionally, we did not receive any direct or indirect compensation related to this study.

### Measures

The primary variables in this study included the NPI. Values for this variable range from 0–5 and were categorized based on the NPI Pupil Reactivity Assessment Scale: “0” indicates a non-reactive pupil with potential severe neurological impairment; 0.1–3.0 indicates abnormal or sluggish reactivity, suggesting possible neurological dysfunction; and 3.1–5.0 indicates normal reactivity reflecting typical neurological function. We assessed GCS scores upon admission to categorize the severity of coma. Secondary variables included demographic data such as age, sex, urine toxicology results, and cause of coma.

The primary outcome measure was ED disposition, specifically whether patients were discharged or admitted to the hospital. For the purposes of this analysis, we included patients who died in the ED in the “admitted” category. The dataset distinctly categorizes patient outcomes as either “discharged” or “admitted,” without any overlap between the two. Consequently, no patients categorized as “discharged” had a recorded death in the ED.

### Data Analysis

We conducted statistical analysis to summarize patient demographics and clinical characteristics. Continuous variables were reported as means and standard deviations or as medians and interquartile ranges (IQR), depending on their distribution. We summarized categorical variables using frequencies and percentages. To evaluate the association between NPI scores and discharge status, we used ANOVA while adjusting for potential confounders. The results are presented as odds ratios (OR) with 95% confidence intervals (CI), and a *P*-value of less than 0.05 was considered statistically significant.

In our analysis, we categorized NPI measurements based on the first recorded measure for each patient, unless it was a non-readable error. This approach was chosen to maintain consistency in our data analysis and to minimize potential bias from selecting higher or lower values from repeated measurements. If the left and right measurements were discrepant, this was noted as it might have been indicative of the cause of coma.

We specified the primary patient outcome as ED disposition, or admission/discharge status. The associations between admission outcome and NPI score categories (primary) and patient demographic characteristics (secondary) were tested using chi-square or Fisher exact tests. We tested associations between outcome and age with the Wilcoxon rank-sum test, and a *P*-value of less than 0.05 was considered statistically significant.

## RESULTS

We assessed 60 patients for eligibility in our study. Of these, 10 were excluded: five did not meet the inclusion criteria; two were initially identified as meeting inclusion criteria but subsequently demonstrated improved mental status, thereby allowing them to verbally decline participation before pupillometer measurements were obtained; and three were excluded due to incomplete data entry. The resultant 50 patients were enrolled in the study, underwent pupillometer measurement, and were included in the final analysis.

After reviewing the medical record for one patient who was initially assigned an NPI score of 0, a second measurement was taken shortly after the first. The first reading was determined to be unreliable, given that an NPI of 0 did not correlate with the patient’s clinical exam, raising concerns for user error. Potential sources of error could include improper device positioning against the patient’s orbit or movement during scanning. Consequently, a second NPI score of 2.3 was obtained and used in the analysis. It is important to note that this patient was ultimately discharged from the ED. Following this adjustment, the updated analysis showed that all 11 patients with an NPI score of 0 were categorized in the admitted group (ie, no patients with an NPI score of 0 were discharged).

The overall median age of patients within our study sample was 58 years (IQR 42–74). Patients who were admitted for further workup had a significantly higher median age of 67 years (IQR 58–80) compared to those who were discharged from the ED, who had a median age of 50 years (IQR 38–72) (*P* = .01) (Table 1). When examining the age distribution of our patients across their respective NPI score categories, the median ages were 63 years (IQR 53–73) for an NPI score of 0, 77 years (IQR 48–89) for NPI score of 0.1–3.0, and 52 years (IQR 40–73) for an NPI score of 3.1–5, with no significant difference found between these groupings (p = .30) (Table 2). Our study enrolled 17 females (34%) and 33 males (66%). Both groups were equally likely to be admitted or discharged (*P* = .30) (Table 1). Stratification by NPI score categories revealed that males comprised 64% of patients with an NPI score of 0, 33% of patients in the NPI 0.1–3.0 group, and 73% of those in the NPI 3.1–5 group. Of note, females comprised a larger proportion of the NPI 0.1–3.0 group (67%) (*P* = .20) (Table 2). Of the 50 patients included in this study, 66% were male and 34% were female. There was no significant difference in admission or discharge status based on sex (*P* = .3). Regarding ethnicity, 18% of patients were of Hispanic/Latino origin, 68% were non-Hispanic/Latino, and 14% were categorized as other/unknown, with no significant differences in disposition (*P* = .20). A more detailed breakdown of sex and ethnicity distributions is provided in Table 2.

When patients were stratified based on GCS scores, we found a significant difference between those who were admitted and discharged (*P* = .02) (Table 1). Most patients with a GCS score of 3 were admitted (84%) compared to those discharged (42%). When stratified by NPI score, 92% of patients with an NPI score of 0 had a GCS of 3, compared to 17% in the NPI 0.1–3.0 group and 52% in the NPI 3.1–5 group. (*P* = .65) (Table 2).

The causes of coma varied significantly between admitted and discharged patients (*P* < .001) (Table 1). Cardiac causes were predominant among admitted patients (74%), while drug overdoses were more common among discharged patients (55%). When analyzed by NPI score, cardiac causes were most frequent in the NPI 0 group (91%), whereas drug overdoses were most prevalent in the NPI 3.1–5 group (47%). Neurological causes were also notable, comprising 9.1% of the NPI 0 group, 17% of those with NPI 0.1–3.0, and 28% of the NPI 3.1–5 group (*P* < .001) (Table 2). Toxicology screening revealed that, overall, 22% of patients tested positive for methamphetamine, 6% for opioids, 16% for cocaine, 18% for benzodiazepines, and 46% for other substances, while 42% had no substances detected (Table 1). There were no significant differences in toxicology screen results between admitted and discharged patients for methamphetamine (*P* = .50), opioids (*P* = > .90), cocaine (*P* = > .90), benzodiazepines (*P* = .50), other substances (*P* = .30), and those with no substances detected (*P* = .20). Stratification by NPI scores showed no significant differences for methamphetamine (*P* = .30), opioids (*P* = .40), cocaine (*P* = .30), benzodiazepines (*P* = .20), other substances (*P* = .80), and no substances detected (*P* = .60) (Table 2).

## DISCUSSION

The advent of pupillometry has paved the way for an objective measure of autonomic nervous system function, a tool with possible high utility in the ED. Our study demonstrates that the NPI is a valuable prognostic tool as it provides a reliable means of assessing the likelihood of patient discharge. The data indicate that higher NPI scores are closely associated with an increased probability of discharge, thereby underscoring the index’s potential as a robust measure for evaluating neurological function in the ED setting. This is particularly relevant in the fast-paced environment of the ED where critical decisions are made rapidly. The consistent performance of NPI across different clinical scenarios (ie, drug overdoses, cardiac issues, and non-traumatic etiologies) in our study highlights the versatility of this tool and has also been reported in prior research examining the stability of pupillometry in dynamic environments.^3^,^12^,^13^

Prior to the development of pupillometry, clinicians mainly relied on traditional methods of assessing neurological function, such as manual PLR evaluations, which use subjective terms like “brisk” or “sluggish” to describe pupillary responses. Studies comparing manual and automated pupillometry have demonstrated poor concordance between the two techniques, wherein manual assessments fail to detect a significant proportion of cases with anisocoria or abnormal PLR responses. In a study by Couret et al, investigators found an 18% overall discordance rate between the two techniques, which increased to 39% for smaller pupils (< 2 millimeters).^5^ Another study by Nyholm et al demonstrated that automated pupillometry had twice the reproducibility and repeatability of manual assessments, highlighting the former’s superiority as an objective and reliable tool.^14^

The NPI offers a standardized, quantitative approach that reduces interobserver variability and enhances the precision of bedside neurological assessments. This observation is mostly supported by research exploring the use of NPI in the ICU setting. A study by Cha et al analyzed the use of NPI in predicting neurocognitive outcomes in patients with acute carbon monoxide poisoning by obtaining initial pupil measurements in the ED.^15^ Their results showcased that NPI was superior to standard PLR using a penlight in predicting one-month neurocognitive sequelae. Among patients in their study sample with a GCS < 12, they found that an NPI < 1 was a highly specific predictor of poor outcomes. These results align with our findings that lower NPI scores are associated with worse clinical outcomes, thereby reinforcing the potential role of NPI as a prognostic tool specifically in ED settings.

The influence of demographic and clinical variables on NPI measurements and patient outcomes is a significant aspect of our study. Factors such as age, sex, and the underlying cause of coma were found to impact NPI scores and, consequently, the likelihood of patient discharge or admission. For instance, older patients with lower NPI scores were more likely to be admitted, while younger patients with higher NPI scores had a greater chance of being discharged. This highlights the importance of considering these variables when interpreting NPI scores in the ED. Additionally, the cause of coma—whether cardiac, drug-related, or neurological—played a critical role in determining outcomes.^4^,^7^ The variation in NPI scores across different demographic groups and clinical conditions underscores the need for a comprehensive approach when using NPI as a prognostic tool. While NPI is a valuable predictor, it should be used in conjunction with a full clinical assessment to ensure accurate prognostication.

The application of NPI in the context of the increasing number of drug overdose cases is particularly pertinent. With the ongoing opioid crisis and the increase in polysubstance use, the ability to quickly and accurately assess neurological function in intoxicated patients is crucial.^8^,^10^ Our study found that NPI remains a stable and reliable measure even in the presence of drug-induced effects such as miosis, a common outcome of opioid use.^9^ This stability is vital for ensuring accurate assessments and appropriate management of intoxicated patients in the ED. By offering an objective measure unaffected by the confounding effects of drugs, NPI enhances a clinician’s ability to make informed decisions for patients experiencing a drug overdose. This application of NPI not only supports timely and accurate diagnosis but also aligns with broader efforts to improve care in emergency settings amid the opioid epidemic.

Our study stands out by focusing on the use of NPI in a broad ED patient population with diverse coma etiologies, rather than limiting the scope to specific conditions such as traumatic brain injury or cardiac arrest. While prior research has explored NPI in ICU settings, our research uniquely demonstrates its feasibility and utility in the ED, a more dynamic and varied clinical environment.^13^,^16^ Unlike studies that concentrate on single conditions, our research covers a wide range of clinical presentations, from drug overdoses to neurological and cardiac causes, providing a comprehensive view of NPI’s applicability across different patient groups. This broader scope both enhances the generalizability of our findings and supports the integration of NPI into standard ED protocols as a clinical adjunct for early neurological assessment and decision-making.

## LIMITATIONS

While the findings of this study are promising, several limitations should be acknowledged. First, the relatively small sample size of 50 patients may restrict the generalizability of our results. To strengthen the validity of these findings, larger studies involving multiple centers are essential. Such studies would help confirm the utility of the NPI in the ED and facilitate the development of standardized protocols for its use. Secondly, the fact that our study was conducted at a single-center, safety-net Level 1 trauma center may have contributed to selection bias. The clinical practices and specific patient population may not be representative of those in other settings, which could impact the applicability of our results to broader contexts. One way we attempted to mitigate this bias was to include all eligible adult patients with a GCS score < 9 and all non-traumatic causes of coma. As a result, we were able to capture a diverse and representative sample of the population seen in EDs.

Additionally, we considered the possibility that certain demographic variables such as age, GCS score, and coma etiology could confound both NPI scores and patient outcomes. To mitigate these effects, our statistical analysis was adjusted to isolate the prognostic impact of the NPI. Specifically, we categorized patients’ first NPI measurement for consistency, and if discrepant left and right pupil measurements were recorded the higher of the two NPI classifications were included in our analysis. Lastly, the observational nature of this study limits our ability to draw causal inferences. We can report associations between NPI scores and discharge outcomes, but establishing causality would require more rigorous study designs such as randomized controlled trials.

## CONCLUSION

This study highlights the effective use of quantitative pupillometry in the ED, demonstrating its potential as a valuable tool for assessing autonomic nervous system function in a more objective and reliable way. The neurological pupil index provides a quantitative measure that enhances the accuracy of neurological assessments, moving beyond the limitations of traditional subjective methods. Our findings suggest that NPI may serve as a valuable adjunct in the assessment of comatose patients in the ED by providing an objective measure of neurological function. However, further multicenter studies with larger sample sizes are needed to validate these findings and establish standardized protocols for the use of NPI in clinical decision-making in the ED.

## Figures and Tables

**Table 1:** Comparison of demographic, clinical, and toxicological characteristics by admission status in comatose emergency department patients.

	Overall N = 50	Admitted n = 19	Discharged n = 31	P-value
Age, median (IQR)	58 (42, 74)	67 (58, 80)	50 (38, 72)	.01
Sex, n (%)				.30
Male	33 (66%)	11 (58%)	22 (71%)	
Female	17 (34%)	8 (42%)	9 (29%)	
Race				>.90
Asian	10 (20%)	4 (21%)	6 (19%)	
Black	13 (26%)	6 (32%)	7 (23%)	
White	12 (24%)	4 (21%)	8 (26%)	
Other/unknown	15 (30%)	5 (26%)	10 (32%)	
Ethnicity, n (%)				.20
Hispanic/Latino Origin	9 (18%)	2 (11%)	7 (23%)	
Non-Hispanic/Latino Origin	34 (68%)	16 (84%)	18 (58%)	
Other/unknown	7 (14%)	1 (5.3%)	6 (19%)	
GCS total, n (%)				.02
3	29 (58%)	16 (84%)	13 (42%)	
4	2 (4.0%)	1 (5.3%)	1 (3.2%)	
5	2 (4.0%)	1 (5.3%)	1 (3.2%)	
6	8 (16%)	1 (5.3%)	7 (23%)	
7	5 (10%)	0 (0%)	5 (16%)	
8	4 (8.0%)	0 (0%)	4 (13%)	
Cause of coma, n (%)				<.001
Cardiac	17 (34%)	14 (74%)	3 (9.7%)	
Pulmonary	1 (2.0%)	0 (0%)	1 (3.2%)	
Drug overdose	18 (36%)	1 (5.3%)	17 (55%)	
Neurological	12 (24%)	3 (16%)	9 (29%)	
Metabolic	2 (4.0%)	1 (5.3%)	1 (3.2%)	
Tox screen positive for				
Methamphetamine	11 (22%)	3 (16%)	8 (26%)	.50
Opioid	3 (6.0%)	1 (5.3%)	2 (6.5%)	>.90
Cocaine	8 (16%)	3 (16%)	5 (16%)	>.90
Benzodiazepine	9 (18%)	2 (11%)	7 (23%)	.50
Other	23 (46%)	7 (37%)	16 (52%)	.30
Tox screen negative	21 (42%)	10 (53%)	11 (35%)	.20

*ED*, emergency department; *IQR*, interquartile range, *GCS*, Glasgow Coma Scale.

**Table 2:** Distribution of demographic, clinical, and toxicological characteristics by neurological pupil index score categories in comatose emergency department patients.

	NPI Score Category			P-value
	0, N = 12	0.1–3.0, N = 6	3.1–5.0, N = 32	
Discharged	0 (0%)	4 (67%)	27 (82%)	<.001
Age, median (IQR)	63 (53, 73)	77 (48, 89)	52 (40, 73)	.20
Sex, n (%)				.20
Male	7 (64%)	2 (33%)	24 (73%)	
Female	4 (36%)	4 (67%)	9 (27%)	
Race				.20
Asian	2 (18%)	4 (67%)	4 (12%)	
Black	3 (27%)	1 (17%)	9 (27%)	
White	2 (18%)	0 (0%)	10 (30%)	
Other/unknown	4 (36%)	1 (17%)	10 (30%)	
Ethnicity, n (%)				.90
Hispanic/Latino Origin	2 (18%)	0 (0%)	7 (21%)	
Non-Hispanic/Latino Origin	8 (73%)	5 (83%)	21 (64%)	
Other/unknown	1 (9.3%)	1 (17%)	5 (15%)	
GCS total, n (%)				.07
3	11 (100%)	1 (17%)	17 (52%)	
4	0 (0%)	0 (0%)	2 (6.1%)	
5	0 (0%)	0 (0%)	2 (6.1%)	
6	0 (0%)	3 (50%)	5 (15%)	
7	1 (8.3%)	1 (17%)	4 (12%)	
8	0 (0%)	1 (17%)	3 (9.1%)	
Cause of coma, n (%)				.001
Cardiac	10 (91%)	1 (17%)	6 (18%)	
Pulmonary	0 (0%)	1 (17%)	2 (6.1%)	
Drug overdose	0 (0%)	3 (50%)	15 (45%)	
Neurological	1 (9.1%)	1 (17%)	10 (30%)	
Metabolic	0 (0%)	0 (0%)	2 (6.1%)	
Tox screen positive for				
Methamphetamine	2 (18%)	3 (50%)	6 (18%)	.30
Opioid	0 (0%)	1 (17%)	2 (6.1%)	.40
Cocaine	2 (18%)	2 (33%)	4 (12%)	.30
Benzodiazepine	0 (0%)	1 (17%)	8 (24%)	.20
Other	4 (36%)	3 (50%)	16 (48%)	.80
Tox screen negative	6 (55%)	3 (50%)	12 (36%)	.60

*NPI*, neurological pupil index; *IQR*, interquartile range; *GCS*, Glasgow Coma Scale.

## References

[1] Olson DM, Stutzman S, Saju C, Wilson M, Zhao W, Aiyagari V (2016). Interrater reliability of pupillary assessments. Neurocrit Care.

[2] Rattan Y, Girgla KK, Prasher P, Magiorkinis E (2023). Recent advances in pupillometry. New Advances in Medicine and Medical Science.

[3] Shoyombo I, Aiyagari V, Stutzman SE (2018). Understanding the relationship between the neurologic pupil index and constriction velocity values. Sci Rep.

[4] Hall CA, Chilcott RP (2018). Eyeing up the future of the pupillary light reflex in neurodiagnostics. Diagnostics (Basel).

[5] Couret D, Boumaza D, Grisotto C (2016). Reliability of standard pupillometry practice in neurocritical care: an observational, double-blinded study. Crit Care.

[6] Thakur B, Nadim H, Atem F (2021). Dilation velocity is associated with Glasgow Coma Scale scores in patients with brain injury. Brain Inj.

[7] Singh P, Stutzman SE, Venkatachalam A (2021). Identification of abnormal pupil dilation velocity as a biomarker of cerebral injury in neurocritically ill patients. Rev Bras Ter Intensiva.

[8] Jung Y (2024). Tracking San Francisco’s drug overdose epidemic.

[9] McKay RE, Larson MD (2021). Detection of opioid effect with pupillometry. Auton Neurosci.

[10] Ruiz-Colón K, Chavez-Arias C, Díaz-Alcalá JE (2014). Xylazine intoxication in humans and its importance as an emerging adulterant in abused drugs: a comprehensive review of the literature. Forensic Sci Int.

[11] Oddo M, Sandroni C, Citerio G (2018). Quantitative versus standard pupillometry for prognostication after cardiac arrest. Ann Intensive Care.

[12] Jolkovsky EL, Fernandez-Penny FE, Alexis M (2022). Impact of acute intoxication on quantitative pupillometry assessment in the emergency department. JACEP Open.

[13] Bower MM, Sweidan AJ, Xu JC (2021). Quantitative pupillometry in the intensive care unit. J Intensive Care Med.

[14] Nyholm B, Obling L, Hassager C (2022). Superior reproducibility and repeatability in automated quantitative pupillometry compared to standard manual assessment, and quantitative pupillary response parameters present high reliability in critically ill cardiac patients. Acta Anaesthesiol Scand.

[15] Cha YS, Ko SB, Go TH (2023). Quantitative pupillary light reflex assessment for prognosis of carbon monoxide poisoning. Front Med (Lausanne).

[16] Oddo M, Taccone F, Galimberti S (2021). Outcome prognostication of acute brain injury using the Neurological Pupil Index (ORANGE) study: protocol for a prospective, observational, multicentre, international cohort study. BMJ Open.

